# The Use and Abuse of Limits of Detection in Environmental Analytical Chemistry

**DOI:** 10.1100/tsw.2008.107

**Published:** 2008-08-08

**Authors:** Richard J. C. Brown

**Affiliations:** Analytical Science Team, National Physical Laboratory, Teddington, Middlesex, UK

**Keywords:** limit of detection, environmental analytical chemistry, performance criteria, measurement method, uncertainty

## Abstract

The limit of detection (LoD) serves as an important method performance measure that is useful for the comparison of measurement techniques and the assessment of likely signal to noise performance, especially in environmental analytical chemistry. However, the LoD is only truly related to the precision characteristics of the analytical instrument employed for the analysis and the content of analyte in the blank sample. This article discusses how other criteria, such as sampling volume, can serve to distort the quoted LoD artificially and make comparison between various analytical methods inequitable. In order to compare LoDs between methods properly, it is necessary to state clearly all of the input parameters relating to the measurements that have been used in the calculation of the LoD. Additionally, the article discusses that the use of LoDs in contexts other than the comparison of the attributes of analytical methods, in particular when reporting analytical results, may be confusing, less informative than quoting the actual result with an accompanying statement of uncertainty, and may act to bias descriptive statistics.

## INTRODUCTION

Limit of detection (LoD) is a commonly quoted performance characteristic of an analytical measurement[1]. Detection criteria are not boundaries in a binary state that indicate the presence or absence of an analyte, but are indications of when a specified probability for the presence or absence of an analyte has been attained[2,3]. The VIM[4] (the International Dictionary of Metrology) defines LoD as the "measured quantity value, obtained by a given measurement procedure, for which the probability of falsely claiming the absence of a component in a material is $\beta$, given a probability $\alpha$ of falsely claiming its presence. IUPAC recommends default values for $\alpha$ and $\beta$ equal of 0.05." Critical decision limits are, by usual definition and practice, based on the distribution of the "blank" signal response. Commonly, the LoD is taken to be three times the standard deviation of the blank response, converted from the intensity produced by the analytical instrument into an analytical quantity by use of the calibration relationship. Practically, this is determined by measuring multiple blank samples. This opinion article is concerned not specifically with the definition or calculation of the LoD, but with two related questions in the context of environmental analytical chemistry:

*The views expressed in this paper are those of the author and do not necessarily reflect the views of NPL Management Ltd.
©2008 with author.
Published by TheScientificWorld; www.thescientificworld.com

1. Should limits of detection be used at all?
2. What constitutes best practice when quoting limits of detection?

## DISCUSSION

### Should Limits of Detection be Used at All?

Having defined the LoD for an analytical process and obtained a series of measurement results, what should be done with values that are below the LoD? There are five possible courses of action for expressing this result[5]. They are:

1. Not detected
2. Zero
3. Less than LoD
4. A fraction of the LoD, such as "LoD/2"
5. The result recorded together with a statement of its uncertainty

What are the benefits and drawbacks (mostly drawbacks!) of each of these approaches[5]? "Not detected" gives us no information at all, not even that the result is less than a certain value, and hence it is not comparable between different laboratories, or over time, as technologies might improve. The use of "zero" is incorrect and misleading (what if the result really was zero!), and can bias descriptive statistics, such as averages and standard deviations, and again gives very little information about the result, offering no comparative value. "Less than LoD" provides some information above the range in which the result lies (between 0 and the LoD), but it does not produce a value that can be used properly in descriptive statistics. Use of a fraction of the LoD, commonly "LoD/2", is arbitrary, but again gives some information about the range in which the results may lie and does yield a value that can be used in descriptive statistics; however, the arbitrary nature of the value produced will bias these statistics. As concluded[5], reporting the value recorded together with its statement of uncertainty provides the most information and does not bias descriptive statistics; moreover, the uncertainty could conceivably be used to produce an uncertainty-weighted average if this was deemed appropriate. (In this way, very low values with high uncertainties would have less weight in descriptive statistics.) This may, however, mean dealing with uncertainties that reach below zero, but guidance is available on this topic[6]. An example of the bias in descriptive statistics that can be caused by the use of these different options for dealing with measurement results below the LoD is shown in Table 1. In the context of the reporting of analytical results, therefore, it is recommended that LoDs are not employed, but the result obtained and its uncertainty quoted instead.

### What Constitutes Best Practice when Quoting Limits of Detection?

Whilst it is clear from Table 1 that, when reporting analytical results, the greatest utility is gained from reporting the results obtained and their uncertainty, the LoD can still be a useful performance parameter when characterising and comparing different methods of measurements, and assessing likely signal to noise performance[7]. When quoting LoD in this context, it is usual to make this statement in terms of the quantity being determined by the measurement procedure. This is usually referred to as the "method detection limit" and is the LoD including all steps of the analytical procedure. For example, suppose a measurement is being made of the mass concentration of chloride in the $PM_{10}$ particulate matter phase of ambient air. This measurement is performed by sampling the $PM_{10}$ onto a filter, extracting the chloride from the collected material into solution, and performing the analytical measurement by ion chromatography. It would seem appropriate to quote the LoD in terms of the quantity and unit in which the

**TABLE 1**
**The Average and Standard Deviation of Eight Measurements***

| Reporting format | Values of eight measurements (LoD = 1) | | | | | | | | Average | Standard Deviation |
|---|---|---|---|---|---|---|---|---|---|---|
| Not detected (ND) | ND | 1.1 | 1.2 | ND | ND | 1.3 | ND | 1.2 | Not defined | Not defined |
| Zero | 0 | 1.1 | 1.2 | 0 | 0 | 1.3 | 0 | 1.2 | 0.6 | 0.6 |
| Less than LoD | <1 | 1.1 | 1.2 | <1 | <1 | 1.3 | <1 | 1.2 | <1.1 | Not defined |
| LoD/2 | 0.5 | 1.1 | 1.2 | 0.5 | 0.5 | 1.3 | 0.5 | 1.2 | 0.9 | 0.4 |
| Result as found | 0.8 | 1.1 | 1.2 | 0.9 | 0.9 | 1.3 | 0.8 | 1.2 | 1 | 0.2 |

* Using an analytical method with LoD = 1, reported using the different criteria discussed in the text.

final measurement is reported[8]; here, nanogrammes of chloride per cubic metre of ambient air, ng.m$^{-3}$. Indeed, this gives readers the best opportunity to compare the attributes of this technique with respect to other measurement methods, and also its likely signal to noise characteristics with respect to likely measured values.

However, on closer inspection, this value alone actually tells us very little. Firstly, the LoD is based on the analytical determination of the mass of chloride on a blank filter. Whilst this quantity remains constant for an unchanging analytical procedure, the relationship between this mass (in ng) and the LoD stated as a mass concentration (in ng.m$^{-3}$) depends on the volume of air sampled. Hence, this method can appear to have an artificially lower mass concentration LoD if a very large air volume is sampled prior to analysis, compared with a small sampled volume, whilst all the time the quantity of chloride in the filter blank remains unchanged. Moreover, the analysis of the chloride in the filter blank has several critical variables associated with it: in this case, the volume of the liquid used to extract the chloride from the filter (and any subsequent dilution to this), the size of the sample loop on the ion chromatograph, and finally, the precision of the ion chromatograph's detector. However, the LoD is critically limited only by the precision characteristics of the detector[9] at the chloride amount level introduced into the instrument during measurement of the blank. Strictly, this would require the LoD to be reported in terms of the mass detectable by the ion chromatograph (in ng), but this is a user-unfriendly approach, making the comparison of different analytical methods more difficult. The absolute value of the LoD, in mass concentration units, is defined by all the other arbitrary parameters associated with the blank analysis. This LoD is then quoted with regard to the volume of air sampled, which is also arbitrary and, moreover, unrelated to the determination of the blank. For example, whilst the process used to determine the blank value might remain constant, if the volume of air sampled prior to analysis is doubled, then the LoD will halve. The situation described for the calculation of the LoD in this example is summarised diagrammatically in Fig. 1.

It is clear, therefore, that to be a useful characteristic, the LoD needs to be quoted with respect to the composition of the analyte in the sample matrix measured. This is the only way in which it can be used to make a useful comparison between unrelated sampling and analysis methods. However, it is important to

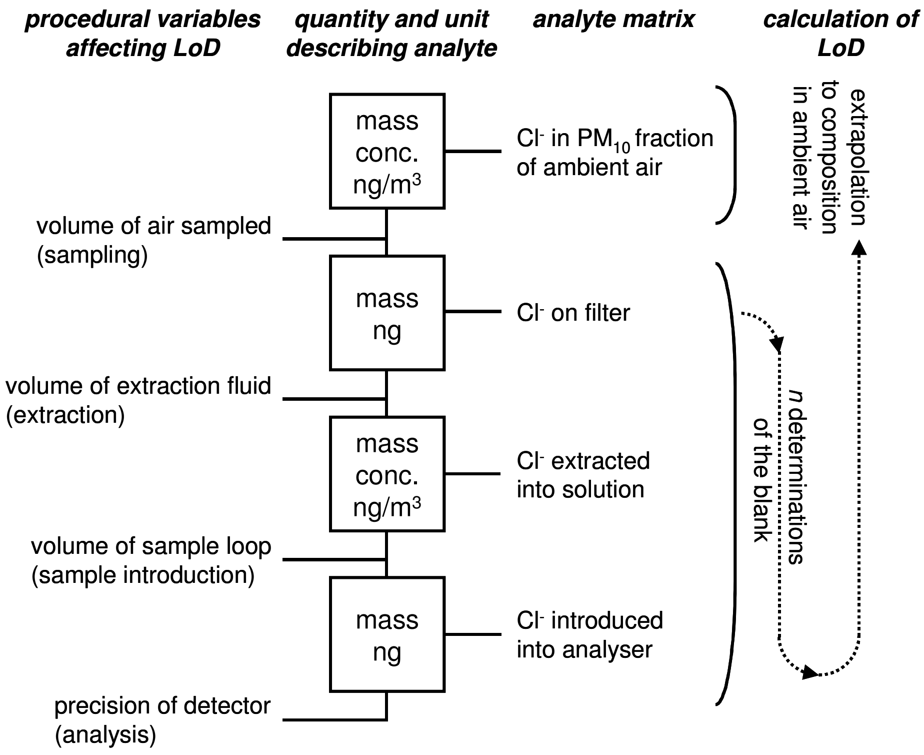

**FIGURE 1.** Diagrammatic representation of the procedural variables and the analytical method processes (in brackets) to which they apply (far left column), affecting the calculation and expression of the LoD for the analysis of the chloride content of the $PM_{10}$ particulate phase of ambient air, and their relationship to the quantity and unit describing the analyte (second left column) and its matrix (third left column) at each stage. A normal measurement process on a real sample would proceed from top to bottom. The column on the far right indicates the direction of flow of the procedure with respect to the determination of the LoD – first the variability of chloride content of the blank filter is measured *n* times to determine a standard deviation for this blank value and then this is expressed in terms of the composition of ambient air by dividing this value by the volume of air sampled.

be aware of the arbitrary nature of this value and, therefore, when quoting such a number, it is essential to list all the parameters that directly affect the calculation of this number, especially those that are not used in the determination of the blank value (such as sampling volume in the example given above). This will usually be done in the experimental description of an analysis, but must be restated when quoting the LoD. Those wishing to evaluate different measurement methods must then take these parameters into account when comparing LoDs between techniques and with respect to the measured levels in real environmental matrices, in order to achieve a true understanding of relative performance.

The different parameters that impose upon the calculation of the LoD are illustrated most clearly when different measurement methods are used to determine the same measurand. Fig. 2 demonstrates this situation for the determination of mercury vapour in ambient air. This shows the different quantities of information required for each measurement technique in order to define the LoD fully in terms of the final measurand quantity (mass concentration in ng.m$^3$) so that the methods can be directly compared. The Zeeman atomic absorption spectroscopy (ZAAS) technique provides a direct measurement of mass concentration and therefore yields the LoD directly by the measurement of the blank. The thermal desorption-atomic fluorescence spectroscopy (TD-AFS) technique measures the mass collected on an Au trap and therefore requires additional knowledge of the sampling volume in order to quote the LoD in mass concentration terms. The inductively coupled plasma-mass spectrometry (ICP-MS) technique is also a measurement of the mass of mercury but, in this case, of the mass introduced into the ICP-MS from the digested Au trap, and therefore requires additional knowledge of the volume of air sampled, the volume of

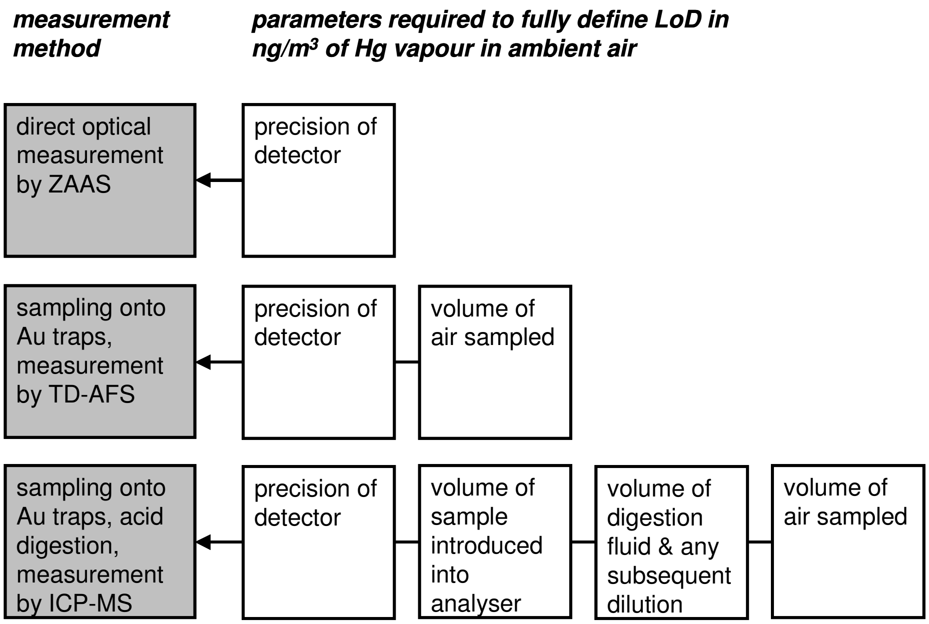

**FIGURE 2.** The parameters required to define and describe fully the LoD for the measurement in ng/m$^3$ of Hg vapour in ambient air by three measurement methods: direct optical measurements by ZAAS, sampling onto Au absorption traps followed by measurement with TD-AFS, and sampling onto Au absorption traps, digestion with acid, followed by measurement with ICP-MS.

the digestion fluid and any subsequent dilutions, and the volume of sample introduced into the ICP-MS, in order to quote the LoD in mass concentration terms. The sampling volume can be increased in order to artificially decrease the LoDs for the TD-AFS and ICP-MS methods, whilst this is not possible for the ZAAS technique.

This second example emphasises that the LoD is critically related only to the precision characteristics of the analytical instrument at the analyte amount level introduced during the blank measurement. Fundamentally, therefore, the LoD may only be improved by acquiring blank material with a lower analyte content (assuming that the analytical instrument in not homoscedastic) or using an analytical instrument with better precision. Changes to other parameters, such as increasing sampling volume, decreasing the volume of the digestion solution, etc., only serve to decrease the LoD artificially.

## CONCLUSION

The LoD serves as an important method performance measure that is useful for the comparison of measurement techniques and to assess likely signal to noise performance. However, the LoD is only truly related to the precision characteristics of the analyser used at the analyte amount level introduced during the blank measurement. Other criteria, such as sampling volume, can serve to distort the quoted LoD artificially and make comparison between various analytical methods inequitable. Therefore, to allow a proper comparison of LoDs between methods, it is necessary to state clearly all of the input parameters relating to the measurement method that has been used in the calculation of the LoD. Moreover, it has been discussed that the use of LoD in contexts other that the comparison of the attributes of analytical methods, in particular when reporting analytical results, may be confusing, less informative than quoting the actual result with an accompanying statement of uncertainty, and act to bias descriptive statistics. Therefore, the use of LoD in this context is not recommended.
